# American Academy of Dental Science

**Published:** 1900-05

**Authors:** 

**Affiliations:** American Academy of Dental Science


### AMERICAN ACADEMY OF DENTAL SCIENCE.

THE regular monthly meeting of the American Academy of Dental Science was held at Young's Hotel, Wednesday evening, December 6, 1899, at six o'clock.

*President Pond.*—It is very evident we have a subject of unusual interest to-night. Whether it is due to the unfortunate case that we have had in the city, and the discussion induced by the fact that it was reported in the public prints, it has attracted a great deal of attention. I have no doubt that this evening we shall learn much more about it than we have previously known. I have to announce the report by Professor Thomas Fillebrown of "A Fatal Case of Hæmophilia."

(For Dr. Fillebrown's paper, see page 302.)

*President Pond.*—We are fortunate in having with us to-night another gentleman who is interested in this case, and had a part in the treatment. I have the pleasure of introducing Dr. Charles A. Porter, who will give us a paper on the subject of "Hæmophilia."

(For Dr. Porter's paper, see page 295.)

DISCUSSION.

*President Pond.*—This is a very interesting and instructive paper. We are remarkably fortunate in having present so many gentlemen who took part in the treatment of this case. It gives me great pleasure to introduce Dr. G. W. W. Brewster, who will open the discussion.

*Dr. G. W. W. Brewster.*—Gentlemen, I think we all feel indebted to Dr. Fillebrown for reporting this fatal case of hæmophilia. If one looks over the literature of the subject, he is impressed by the number of successful cases reported in the various journals, and of the different methods of stopping bleeding in hæmophilia. In the light of Dr. Porter's paper, it seems to me probable that many of these cases were not true hæmophilia.

In reporting a fatal case like this one of Dr. Fillebrown's, a surgeon or a dentist shows a great deal of courage. As a rule, we do not like to report our fatal cases, but it is from the report of just such a case as this that we obtain, perhaps, some of our most useful knowledge.

I remember very well a case that Dr. Richardson operated on for tumor of the kidney, in which the patient gave a history of hæmophilia,—*i.e.*, of excessive bleeding,—from a cut at some past time during her life. I should not say a history of hæmophilia, but a history of bleeding, for I do not believe it was hæmophilia. The operation was justified by the severity of the case, and the tumor of the kidney was removed. The patient made a perfectly good recovery. If this had been hæmophilia, there would have been a complication of hemorrhage.

I cannot speak from any extended personal experience of this disease, because Dr. Fillebrown's case was the first one I had ever seen. In dealing with hemorrhage in surgery, our methods are almost always successful—I should say, always successful—in connection with operations. If large wounds are left, and the oozing cannot be controlled by the pressure of forceps and ligating the vessels, a firm pressure of gauze almost invariably controls the hemorrhage. The advantage of gauze over all other material for pressure lies in the fact that a firm pad of gauze over a bleeding surface affords pressure on the vessels, while it allows the blood to escape into the meshes, and there is no chance for a layer of clot to form between the dressing and the bleeding surface. This, I think, is the reason that absorbent gauze is better to use for hemorrhage than absorbent cotton.

Dr. Porter has covered very fully the medical aspects of the subject, and I have nothing further to add. From a surgical point of view I think it might be interesting to show you the method and the apparatus necessary for salt infusion. In the last part of his paper he stated that salt infusion—*i.e.,* the introduction of a salt solution into the vessels—was probably of no use. And, theoretically, it seems as though that were perfectly true, because if we inject a quart of salt solution, it would probably run out as freely as the blood. It seems to me, however, that there is a time when salt infusion might be of service in these cases. Suppose that all methods have failed, and we are waiting for the time to come when the patient has bled all that he can bleed, hoping that the hemorrhage will cease just before he stops breathing: salt infusion at that critical time might give enough renewed strength to tide him over, and with renewed attempts to stop the bleeding the fatal outcome might be warded off.

There are two methods of introducing salt solution into the organism in cases of excessive loss of blood. One is by infusion under the skin, into the subcutaneous tissue. The method is very simple, and does not rank as an operation. It requires simply a fountain syringe, a large-sized hypodermic needle, and a sterilized salt solution, which is boiled water with six-tenths of one per cent. of salt in it. The needle is put under the loose skin of the flanks or breast, and the fountain syringe raised high enough to allow the water to run slowly into the subcutaneous spaces. Between a pint and a quart of water can be introduced in that way, supplying fluid for the emptied vessels. For immediate and quick effect, the infusion into the veins is the most satisfactory method. In cases of great loss of blood, when the patient's pulse has become thready and uncountable at the wrist, a pint or more of salt solution injected into the vein will revive him.

I have brought with me the apparatus necessary for the salt infusion. The operation is very simple, and can be performed, I am sure, by any man who is as dexterous with his hands as a dentist. This particular kind of a canula, which has a blunt point, is necessary, because if it has a sharp point it may puncture the other side when it is put into the vein. It has this rubber tube with a funnel at the end. This can be boiled before operating. The place of infusion is usually a vein of the arm, one of the veins at the bend of the elbow, the one that stands out the most. A bandage is placed around the arm just above the elbow. This fills the veins and shows the most prominent one at the bend. An incision is made through the skin, and the vein dissected from the surrounding tissues. A space of at least half an inch of the vein should be exposed. Then a piece of silk or any form of ligature is placed around the upper part of the vein, and another piece of silk below. The bandage is removed from above, and the ligature below is drawn up. This holds the vein up out of the wound, so that a small cut can be made in it between the ligatures. The apparatus in the mean time is filled with the salt solution and the water allowed to run out of the end of the canula to remove all air. With the salt solution dribbling out of the end of the canula, the incision is easily found and the point of the canula is slipped into the vein. The ligature is tied around the canula, and the salt solution passes into the circulation. The usual amount given at one time is between a pint and a quart, and the temperature is about 100° F. The hotter the solution, the more quickly it will affect the circulation. The effect is instantaneous on the pulse.

I am sure there are many gentlemen here who have had cases of hæmophilia, and can add their experiences to this discussion. I thank this society for the invitation to be present this evening.

*Dr. M. C. Smith.*—There is one feature that has not been touched upon, and that is septicæmia. These patients, after they have bled for eight or ten days, are very susceptible to septic influences; and I have seen more uneasiness, or more bad symptoms, from what you would be more inclined to call septic symptoms than from loss of blood.

There are a couple of cases that I would like to report this evening. One was not a patient of mine, but I was consulted in regard to it. Mrs. A. was taken to a hospital with a slight laceration of the cervix. Dr. P., a very careful surgeon, operated. After he had stitched the parts together he found there was still an excessive hemorrhage. The more stitches he put in, the worse the hemorrhage became, and he had to resort to a very firm vaginal tampon to control that hemorrhage. It caused a deal of trouble. The patient finally rallied and picked up. Soon after leaving the hospital she went to one of our fellow-practitioners in the city and had some teeth out. She bled for a number of days, until she was in rather a septic condition. She had also an eruption, especially on the thighs. A doctor in Maine told her the dentist had inoculated her with syphilis. Her husband came back to Lynn very much wrought up over the subject, and went about consulting a number of physicians and lawyers as to what the legal aspects of the case might be. He went to Dr. P., who told him that he could see no indication of syphilitic contamination. The husband vowed that his doctor in Maine claimed it was syphilis, and he was going to sue that dentist and have satisfaction. Dr. P. said to him, "Young man, you had better go a little slow; it will bother you considerably to prove that eruption is syphilitic, and then some of your neighbors may accuse you of giving the syphilis to your wife yourself." I believe that ended the legal aspect of that case.

I now wish to report on a patient of mine, a little girl. From birth up to somewhere about three years of age the parents had little hope of raising her. This same Dr. P. was called in, and he kept her along for a number of years, and the "sheet anchor" of his treatment was ergot; that was the only thing that seemed, in his opinion, to do any good. She came to me probably about the sixth or seventh year, to have some temporary teeth extracted. The story, as told by the mother, was that she would bleed for days at a time from the nose, also from the ears and eyes. When a cold sore on the lip was about ready to scale off, a little crack would start in and bleed sometimes a week or ten days at a time. I extracted teeth several times, and I think, without any exception, she bled for ten days constantly. In that case I did not dare to use a compress. I found that the more pressure I used, the worse it was, and the blood would ooze out right at the edge of the pressure. If I exerted pressure over the bleeding portion, it was sure to start up a worse hemorrhage than that which I was trying to control. In that case I gave up pressure entirely. The best results I had were from light applications of the solution of persulphate of iron, applied in rather diluted strength, but we had to depend principally upon ergot; and in her case this worked very nicely indeed. The mother always kept it in the house, and whenever the girl had these spells, she commenced giving her the ergot.

Several years after, I extracted all of her four first molars, and each time we got the regular ten or twelve days' hemorrhage. She kept on in this manner until she was about thirteen years of age, when she had her first menstruation, and came very near dying at that time. Dr. P. was called in, and found her in a very critical condition. He looked her over quite carefully, and the conclusion he came to was, that it was useless to use a vaginal tampon. He said if he did, the pressure he would have to use to get it in solid enough to do any good would in all probability have ruptured some of the hymeneal arteries, and he was afraid he would get a worse arterial hemorrhage than from the menstrual bleeding. His treatment was complete rest, lying on the back, head down. After some days she rallied from that. She picked up wonderfully, and looked as healthy, bright, and strong as any young girl I have seen for some time. After the first menstruation I had her as a patient, for some filling. She was in the office a number of times, and I remarked how well she looked. At her second menstruation she died from loss of blood. I do not think in that case there were any septic symptoms whatever, but that she died from exhaustion due to loss of blood.

*Dr. Eames.*—Gentlemen, in speaking further upon this question, it may be mentioned that it is possible to induce a condition similar to hæmophilia in previously healthy persons by the privation of fresh air and sunshine. I once had a patient who was living in this unhygienic condition, and the extraction of a tooth was followed by hemorrhage lasting about a week, although the usual treatment was employed. One year from that time a similar tooth on the other side of the mouth was removed, but on this occasion the fluid extract of ergot was administered for one week previous to the operation, and nothing more than the normal amount of hemorrhage followed.

As to the local styptic, I think a good many would disagree with the use of persulphate of iron on account of the irritation which might ensue. In any case of protracted hemorrhage it would hold quite as good as in a case of hæmophilia. I do think that the manner of using the local styptic is important. Tannic acid should be preferred to the iron, as it appears to form a stronger and firmer clot. A very small piece of cotton saturated with tannic acid and glycerin should be carried to the extreme bottom of the alveolus, followed by larger pieces.

I have no personal experience with the use of the galvanic current for the purpose of stimulating contraction of the arteries, but it has been found useful.

It seems important to take note of the action of the heart in cases of severe hemorrhage, for there is, necessarily, extreme action of the heart on account of the nervous excitement and loss of blood. For this condition I would suggest the use of opium; and the acetate of lead to promote coagulability of the blood.

*Dr. Daly.*—It has been my unfortunate experience to have to extract many teeth in the last few years, and I cannot but note that in the last ten weeks I have had more hemorrhagic cases than in any ten years all put together. I have the record of eleven that have taken place in the last ten weeks, all of more or less severity; nothing like intense hæmophilia, however, but they were very annoying bleeding cases. I have always used for many years ammonio-ferric alum, and have had fair results. I would like to ask the gentlemen if they have noticed any cases of bleeding lately, more than at any other time?

*Dr. G. T. Baker.*—The very day that I received the card of the Academy announcing the subject for this evening,—"Hæmophilia,"—a young man presented himself and informed me that he was a bleeder. It was the first time I had ever had such a case, and so I went to work with unusual care. There was one tooth that had an exposed pulp, and I made an application to destroy the pulp; and it being an approximal cavity, I allowed the dressing to rest on the gum slightly and to remain there several days. When the patient came back I noticed a slight oozing of blood, not only from where the cotton touched the gum, but for quite a little distance beyond, and, in fact, he told me it had been bleeding. I removed the dressing and did nothing, and observed next time that the case was all right.

There is one remedy for secondary hemorrhage which I heard of a great many years ago, and which I have not heard spoken of in a dental meeting since. I have had occasion to use it several times, and it has always worked very satisfactorily. I used it in one case where a young man had bled for several days, and the hemorrhage stopped at once. It consists of picric acid, and it may be familiar to many here. It is made by taking a drop of creosote or carbolic acid and a drop of nitric acid, put side by side on the top of an inverted glass or other convenient article. Now take a pledget of cotton and dip it first in one and then in the other, and after waiting a moment, pack securely in the alveolus. The union of the creosote, nitric acid, and cotton forms a violent explosive compound, and must be handled with care. I think Dr. Daly is familiar with this method. Dr. I. J. Wetherbee first taught it at the Boston Dental College, and I think the method is original with him; in fact, I am sure it is—he told me so.

*Dr. Joslin.*—There are two points that interested me especially: one was that hæmophilia was extremely rare in women; in fact, being twelve times as common in men. In this connection the statistics of the Massachusetts General Hospital in gastric ulcer may be mentioned. In the last ten years, 1888-1898, one hundred and eighty-seven cases of this disease were treated in the hospital. Dr. Greenough and I found that mortality from hemorrhage was seventeen per cent. among the men, but only one and twenty-seven-one-hundredths per cent. among the women. Not a single woman died of hemorrhage under the age of thirty.

The second point is the use of a remedy which I think you will find mentioned more and more this coming year in the medical journals. Whether it will be of value is certainly questionable, as is the case with all such remedies, but it is well worth your attention, because several very good men have spoken favorably upon it. It is the use of a solution of gelatin in cases of aneurism and hemorrhage. It has been tried—not very successfully, I think I may say—in this country in aneurism. The remedy has been employed to check hemorrhage in cases of bleeding from the stomach, and also in cases of bleeding from other organs. Furthermore, by one man it was reported as successful in a case of melæna neonatorum. I have simply mentioned it because I am positive that the remedy will be tried a great deal in the next few months, and I am sure you will be interested to watch the reports as they come out.

*Dr. Brackett.*—I should be obliged if the gentleman would make it a little plainer as to the method of application of this medical agent,—whether it is local, or how applied.

*Dr. Joslin.*—Originally these injections of gelatin were given under the skin, and a two-per-cent. solution was used, especially in the cases of aneurism; but a great deal of pain ensued, and consequently a one-per-cent. solution was substituted. In other cases Dr. Paljakow gave the solution by the mouth and rectum; and in the case of melæna neonatorum a ten-per-cent. solution of gelatin was made on the spot and given by enema.

*Dr. Brackett.*—This is an aqueous solution?

*Dr. Joslin.*—Yes, an aqueous solution. The amount given was from two hundred to two hundred and fifty centimetres.

*Dr. Briggs.*—I have no right to speak on this subject, as I have never seen a true case of hæmophilia, but I have some bleeders, and can speak of the treatment in those cases in one or two instances that seemed to work rather favorably.

I do not believe in the iron solutions. It seems to me that they always irritate, and are very apt to be followed by still greater bleeding. And I do think that what has been said about rest, and particularly about a low diet, is a very important part of the treatment, and also the avoiding of any actual pressure. I have always done a great deal with antiseptic sponge, which is very much in the line of the gauze and has the same purpose, but I think it acts better than gauze. I speak particularly from the dentist's standpoint, in treating the sockets of teeth, and for that purpose the bit of sponge that is fitted to the shape of the socket—not to be larger, not to expand, not to crowd the socket, but fitted to put into the socket—has worked admirably in many cases.

And also, in condemning the iron solution, one ought to put something forward to take its place, and it seems to me that chloride of aluminum is an astringent that ought to be more widely appreciated than it is. I have recommended it to several surgeons, who have used it for hemorrhage where nothing else controlled it, and this has succeeded. A neutral solution of chloride of aluminum can be used in various strengths, and is very astringent, and non-irritating in that it does not produce inflammation. Of course, in strong solutions it is irritating, producing smarting, but it does not produce subsequent inflammation.

*Dr. E. H. Smith.*—As plaster of Paris has not been spoken of in the discussion, I would speak of its use in connection with the mechanical treatment for excessive bleeding. Take a pledget of cotton, saturate it with a styptic,—I commonly use tincture of kino,—and carry it into the socket; then saturate a pledget of cotton with plaster of Paris, and carry that in. Then take a part of an ordinary impression cup, such as we use for modelling compound. Fill that with plaster of Paris, and carry it down over the socket of the extracted tooth. This makes a perfect adaptation to the wound, the gum tissue, and the teeth about it.

I remember, when I was a student, of my preceptor, Dr. Shaw, treating a case in this way, with this exception, that he did not use the cup. He took a cork and fitted it to straddle the socket; then took a pledget of cotton, saturated it with tincture of kino, and carried it well into the socket; placing over this another pledget of cotton saturated with plaster of Paris, and dipping the cork in plaster, carried it over all, and then put on the bandages. There was no further trouble.

I think that much depends upon the mechanical stopping of the wound so there is no outlet. This done, and there is no hemorrhage through the tissues, we have accomplished a cure.

*Dr. Williams.*—I am glad to hear Dr. Smith mention that point, external pressure, for that has been one of the most successful means by which I have met these cases,—the external pressure as well as the internal. The interior pressure sometimes gives a chance for the blood to ooze out, but put on the external like a clamp, and you keep it perfectly tight.

The plaster of Paris also is a good suggestion. I think it was first mentioned by the late Dr. Buckingham, of Philadelphia, several years ago. I heard him speak of having been very successful with it. I have tried it in some cases successfully, but this very essential point I wish to emphasize,—the necessity of the external pressure as well as the pressure in the socket.

*Dr. Eames.*—The extreme tendency to bleed in cases of hæmophilia is shown in the hemorrhage breaking out in other places after having been successfully controlled from the original source, hemorrhagic spots appearing upon different parts of the body.

*Dr. Werner.*—The chloride of aluminum is sold to dentists by dealers under the name of mauroline, and I wish Dr. Briggs had spoken of this, for I find many dentists evidently do not know it.

*Dr. Brackett.*—In an experience of more than a quarter of a century I have seen a considerable number of cases of serious bleeding following tooth extraction, but none approaching in severity the case reported by Professor Fillebrown. All my cases have been controlled with comparatively simple, non-irritating astringents, like tannic acid, in connection with comprehensive compression, persistently applied.

When these emergencies arise, the services of the dentist are among the most important which he can render. In numerous instances he saves the patient from very grave danger, not to say fatal results.

It is a curious expression of human nature that for this service, often demanded at the most unseasonable hours and under inconvenient circumstances, there seems in most instances less disposition to make any acknowledgment or compensation than for anything else which the dentist does.

*Dr. Werner.*—I look at the question a little differently. I think it is good instruction for us not to pull out the tooth. I am happy to say I do not have to extract many teeth.

*Dr. Fillebrown.*—I have been pleased to listen to a discussion so generally engaged in. It is worth all that our friend, Dr. Brewster, implied in speaking of the courage on the part of the operator in reporting the case being commendable. I certainly think it is worth all it cost me to be the cause of so interesting a discussion as this.

The sockets of the three roots were entirely plugged by coagulum. There was no oozing of blood from the sockets, and consequently any interference with them was unnecessary and was not attempted. The bleeding was on the edge of the gum opposite the root of the first molar. And I am quite sure that there was very little wounding of the tissue, as the roots were loose, and it is difficult for me to account for the fact that this one spot should have bled as it did.

I have never seen any ill results or any secondary bleeding from the use of persulphate of iron, and I have always considered it my right bower. While alum and other mild astringents answer well in perhaps a great majority of cases, yet when I need something that will do sure work, I always feel that I have in iron a resource that the milder astringents do not afford. It not only affects the blood and coagulates what comes in contact with it, but has a marked effect upon the terminal nerves, and causes a contraction of the terminal arteries.

I did not fully understand what Dr. Porter intended to convey by the "degeneration of the arteries" in the case of hæmophilia, whether or not it takes place in the short time that the bleeding continues, say, in the six days that intervened in this case between the operation and the end?

*Dr. Porter.*—Yes, I mean that in the six days, where the hemorrhage has been very severe and the person is suffering from anæmia, you get a very rapid fatty degeneration, especially in the cells. I do not mean that the arteries become actually thinner.

*Dr. Fillebrown.*—During the last few weeks I certainly have observed the conditions remarked by Dr. Daly and also a great amount of inflammation. Old teeth that have long been resting quietly have suddenly become inflamed and often an abscess has formed. Last Saturday I extracted a third molar for one of our medical friends. This patient was in no sense a hæmophiliac, but the next day he telephoned to me that it had been oozing blood all through the night. When I arrived there about noon it was still oozing a little. I removed the packing; it was somewhat colored. I washed out the wound with some hot water, and fortunately the bleeding stopped. It is very evident that trouble is in the air about this time of the year.

CHARLES H. TAFT, D.M.D.,
*Editor American Academy of Dental Science.*

